# Comparative study of triage strategies for women with atypical squamous cells of undetermined significance in the post-vaccine era

**DOI:** 10.3389/fonc.2024.1416116

**Published:** 2024-10-08

**Authors:** Hongmei Yang, Yubing Hao, Meili Niu, Jie Zheng, Xinhua Jia, Shaokai Zhang, Libing Wang, Xun Zhang, Qinjing Pan, Xiangxian Feng, Youlin Qiao, Zhifang Li

**Affiliations:** ^1^ Department of Public Health and Prevention, Changzhi Medical College, Changzhi, China; ^2^ School of Public Health, Shanxi Medical University, Taiyuan, China; ^3^ Department of Epidemiology, National Cancer Center/National Clinical Research Center for Cancer/Cancer Hospital, Chinese Academy of Medical Sciences and Peking Union Medical College, Beijing, China; ^4^ Department of Cancer Epidemiology, Affiliated Cancer Hospital of Zhengzhou University, Zhengzhou, China; ^5^ Department of Pathology, Affiliated Heping Hospital of Changzhi Medical College, Changzhi, China; ^6^ Department of Pathology, National Cancer Center/National Clinical Research Center for Cancer/Cancer Hospital, Chinese Academy of Medical Sciences and Peking Union Medical College, Beijing, China; ^7^ Department of Cytology, National Cancer Center/National Clinical Research Center for Cancer/Cancer Hospital, Chinese Academy of Medical Sciences and Peking Union Medical College, Beijing, China; ^8^ Center for Global Health, School of Population Medicine and Public Health, Chinese Academy of Medical Sciences and Peking Union Medical College, Beijing, China

**Keywords:** human papillomaviruses, atypical squamous cells of undetermined significance, cervical cancer, resource-limited areas, the post-vaccine era

## Abstract

**Objective:**

The research focused on a comparative analysis of triage strategies for women with Atypical Squamous Cells of Undetermined Significance (ASC-US) before and after receiving the HPV vaccine, aiming to optimize cervical cancer prevention strategies, especially in resource-limited healthcare settings.

**Materials and methods:**

Between September 2018 and December 2023, 7,511 women aged 21 years or older who underwent liquid-based cytology for cervical cancer screening were recruited. Women diagnosed with ASC-US were included in the study. All participants underwent HPV testing and liquid-based cytology examination, and those with abnormal results were referred for colposcopy. Women with abnormal colposcopy findings underwent further histopathological examination. The gold standard for diagnosis was pathological, with cervical intraepithelial neoplasia grade 2 or higher (CIN2+) on histology as the endpoints. In the final analysis, 933 women with ASC-US were enrolled as the unvaccinated group, with 179 of them testing positive for HPV 16/18. Assuming that all women would receive the bivalent vaccine targeting HPV 16/18 in the post-vaccine era, and given that the vaccine protection rate is 100% against HPV 16/18, then 754 women excluding those of HPV 16/18 positive would comprise the vaccinated group.

**Results:**

In the unvaccinated group, the overall HPV positivity rate was 59.27% among ASC-US women, with a 100% HPV prevalence rate among those with CIN2+ lesions. The combination genotyping model of HPV16/18 showed the highest specificity (81.77%) and the lowest referral rate (32.37%). In the vaccinated group, the HPV positivity rate was 49.61% among ASC-US women, with a 100% HPV prevalence rate among those with CIN2+ lesions. The specificity of HPV33/58 was the highest (86.99%), and the colposcopy referral rate was lowest (27.54%), with statistical significance. Sensitivity, positive predictive value, and negative predictive value were not statistically significant.

**Conclusion:**

HPV16/18 demonstrated a more efficacious triaging effect in the unvaccinated group. HPV33/58 will potentially replace HPV16/18 as the priority screening genotyping among vaccinated populations.

## 1 Introduction

Cervical cancer is the most common malignancy among female reproductive tract tumors, posing a significant disease burden, particularly in areas with limited health resources (1, 2). Screening for cervical cancer primarily relies on cytology tests and HPV testing. ASC-US, an important cytological diagnosis in cervical cancer screening, is not definitive, and its histopathology results can range from inflammation and cervical intraepithelial neoplasia (CIN) to cervical cancer. Approximately, 3%-10% of women are diagnosed with atypical squamous cells of undetermined significance (ASC-US) (3). The interpretation of cytological results can be influenced by the skill level of the physician, leading to a degree of bias. Over the past period, the integration of HPV testing into clinical practice, including HPV mRNA and HPV DNA testing, has significantly improved the management of ASC-US cases, with HPV testing (4–6). More precise triaging of ASC-US women is crucial for cervical cancer prevention, especially when implementing a stratified management approach tailored to different high-risk human papillomaviruses (HR-HPV) types.

HPV vaccines are the most effective primary prevention measure against cervical cancer (7). The bivalent vaccine offers a protection rate exceeding 95% (8–11), while it is significantly less expensive than the quadrivalent and nine-valent HPV vaccines. Despite this, vaccination coverage remains relatively low in many developing countries (12, 13). Considering the balance between cost and preventive effectiveness, the bivalent HPV vaccines is recommended for the general population in limited health resources settings. In China, National People’s Congress deputies and health experts have called for inclusion of domestically produced bivalent HPV vaccines in the national immunization program to enhance accessibility and affordability for the eligible population.

With the gradual popularization of the HPV vaccine, we will eventually enter the post-vaccine era, where vaccinated and unvaccinated women will coexist for an extended period, and the types of HPV infections will also change. Currently, follow-up data from real-world studies on the HPV-vaccinated population are not readily available or lacking in resource-limited settings, particularly for those with ASC-US. As a result, the specific gene combination that best triages the ASC-US population in the post-vaccine era is rarely reported, the differential triage strategies for ASC-US women who are vaccinated and unvaccinated are worth exploring. In this study, based on an earlier large real-world population undergoing cervical cancer screening, we make the hypothetical assumption that in the future, all women who were initially unvaccinated against HPV have subsequently received the HPV bivalent vaccine. Under this assumption, the subgroup of these women who are not HPV 16/18 positive were considered as the ‘vaccinated group’ for the purpose of our analysis. By comparing the triage efficacy of the vaccinated and unvaccinated groups, we identified different management approaches for ASC-US women in the post-vaccine era in countries with limited healthcare resources.

## 2 Materials and methods

### 2.1 Study design and participants

Since 2009, the “Two Cancers Screening” program for rural women has been implemented in China. This project provides free or subsidized screenings within the rural female population to enhance women’s health status and reduce the incidence and mortality rates of cervical cancer and breast cancer. The cervical cancer screening used co-testing with cytology and HPV testing. This cohort study was based on the “Two Cancer Screening” program in Wuxiang County, Shangdang District, and Zezhou County, Changzhi City, Shanxi Province. Women diagnosed with ASC-US, aged≥21 years, and with sexual experience were included in the study. Exclusions were: 1) pregnant women or women within 8 weeks after delivery; 2) women with a history of hysterectomy, cervix surgery, or cervical cancer treatment; 3) women with cognitive impairment.

**Figure 1:**
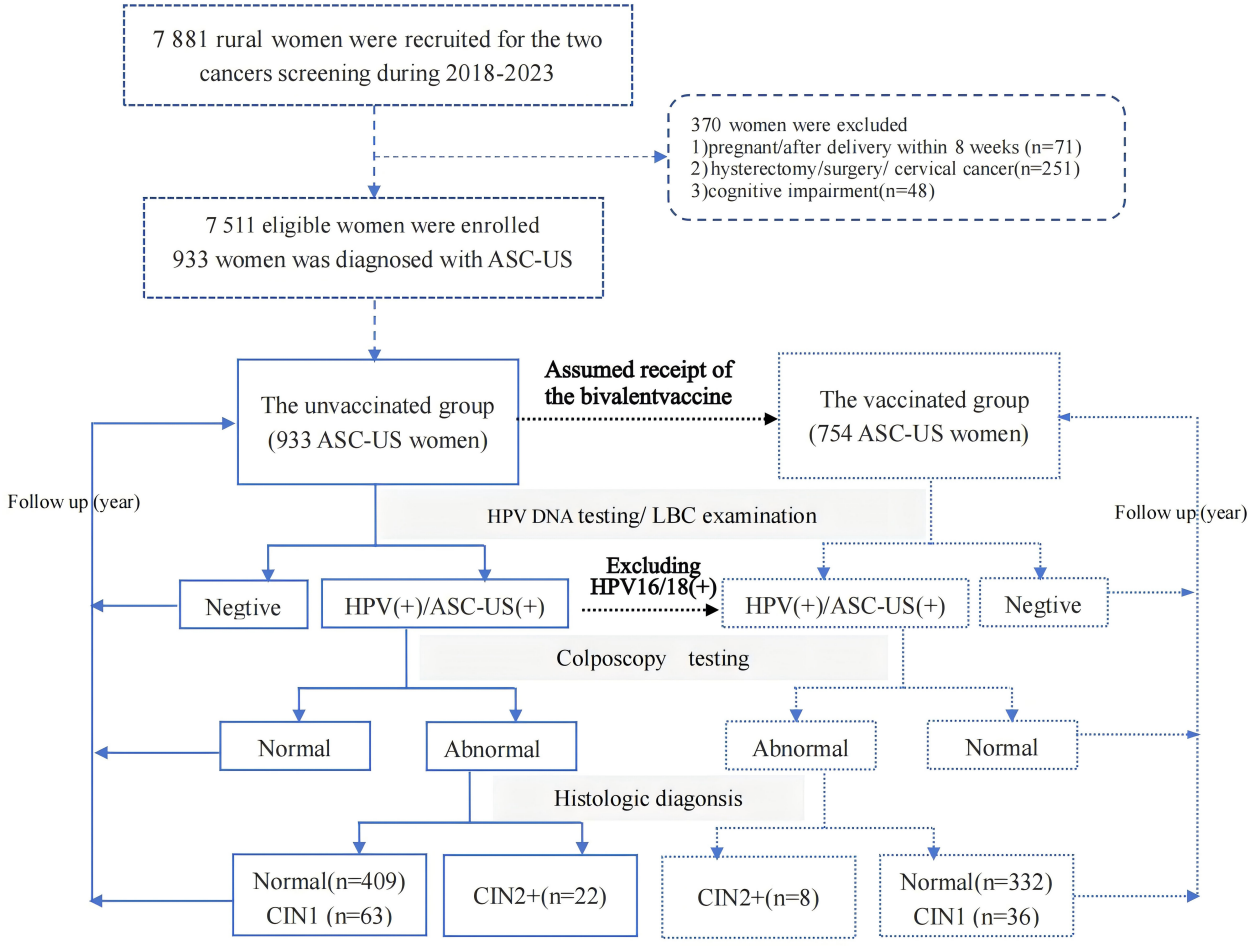
The flowchart of subject enrollment in this study.

Of the 7,511 women enrolled from 2018 to 2023 for cervical cancer screening, 933 women diagnosed with ASC-US were categorized as the unvaccinated group. Assuming that all women in the unvaccinated group would receive the bivalent vaccine targeting HPV 16/18, and given that the protection rate of this bivalent vaccine is 100% against HPV 16/18, the subgroup of 754 women who excluded 179 HPV 16/18 positive women were considered as the vaccinated group. Additionally, 754 women were included as the bivalent vaccinated group, which excluded 179 HPV 16/18-positive women from the 933 ASC-US women. All included ASC-US women were followed up for the next 3 years with HPV DNA testing and liquid-based cytology (LBC) examinations. Women who tested HPV-positive or had ASC-US or and higher results were referred for colposcopy. Those with abnormal colposcopy findings underwent further histopathological examination. Pathological diagnosis was the gold standard, with cervical intraepithelial neoplasia grade 2 or higher (CIN2+) as endpoints. The screening flowchart is shown in **Figure 1**.

### 2.2 Data and specimen collection

Demographic information was collected through questionnaires, including marital status, education level, smoking and alcohol consumption history, menstrual history, and reproductive history. Trained gynecologists conducted gynecological examinations of the vulva, vagina, and cervix for all participants, and speculum examinations were also performed. The specimens of cervical exfoliated cells were collected for liquid-based cytology (LBC) classification and HPV genotyping tests.

### 2.3 Laboratory testing

#### 2.3.1 HPV testing

A commercial assay was used for HPV DNA testing. The HPV testing method was the Biochip Method, manufactured by Beijing Bohui Innovative Optoelectronic Technology, with approval from the China Food and Drug Administration (CFDA) (registration certificate no: 20163401108). This method can detect 14 types of HPV DNA (16, 18, 31, 33, 35, 39, 45, 51, 52, 56, 58, 59, 66, and 68) from the cervical exfoliated cells, and distinguish all HPV types individually. Quality control probes and detection probes are distributed on the hybrid membrane of HPV nucleic acid detector. The quality control probes include blank, negative, color rendering, and internal reference quality control points. The positive quality control is used to verify the validity of the detection method, while the negative quality control is used to exclude the possibility of false positive results.

#### 2.3.2 Cytology examination

Cytology slides were reviewed by two pathologists, and results were reported according to the Bethesda 2014 classification. The cytological results included: negative for intraepithelial lesion or malignancy (NILM), atypical squamous cells of undetermined significance (ASC-US), low-grade squamous intraepithelial lesion (LSIL), atypical squamous cells-cannot exclude high-grade squamous intraepithelial lesion (ASC-H), high-grade squamous intraepithelial lesion (HSIL), atypical glandular cells, and cervical cancer cells. Diagnoses were reported if the diagnoses by two cytologists were consistent. Otherwise, a third cytologist was consulted.

#### 2.3.3 Cytology and histology

All women with positive HPV results or abnormal cytology (ASC-US or worse) were referred for colposcopy. If the colposcopy provided full visibility and a lesion was identified, a biopsy was performed on the abnormal area, with the specific location of the specimen clearly marked. If the colposcopy exposure was insufficient, cervical curettage was performed. Two pathologists independently made diagnoses, if the diagnoses were concordant, they were reported as the pathological diagnosis. Otherwise, a third pathologist also reviewed all positive results and 10% of negative slides. The final diagnosis was based on the agreement between the three doctors, and in cases of disagreement, a consensus decision was made by all three. According to the 2014 WHO Classification of Tumors of the Female Genital Tract (14), histological diagnoses of cervical lesions were categorized as normal, LSIL/CIN1 (including the condylomatous variant), HSIL/CIN2, HSIL/CIN3 (including adenocarcinoma *in situ*) and carcinoma (squamous cell carcinoma or adenocarcinoma).

### 2.4 Quality control

Investigators, gynecologists, and pathologists were trained according to a standardized manual of operation. All technicians, cytologists, and pathologists involved in HPV testing and cytology slide reading were blinded throughout the study. Experienced physicians conducted gynecological and colposcopy examinations. Pathologists with more than 30 years of experience provided the final decisions for cytological and pathological diagnoses. HPV detection probe and quality control probe be used throughout the whole process of HPV detection, and quality control probe be distributed on each chip. Positive and negative quality controls were implemented to ensure the quality of HPV testing.

### 2.5 Statistical analysis

SPSS version 20.2 (IBM Corp, New York, USA) was used for data analysis. Quantitative variables were expressed as medians and interquartile ranges, while categorical variables were represented by numbers and percentages. The pathological diagnosis served as the gold standard, with CIN2+ on histology as the endpoint. A receiver operating characteristic (ROC) curve was plotted, and the sensitivity, specificity, positive predictive value, negative predictive value, area under the curve (AUC) of the ROC, and referral rate of HPV genotyping were calculated. The referral rate was calculated as the number of participants with ASC-US and positive HR-HPV dividing by the total number of participants with ASC-US. The chi-square test and Fisher’s exact probability test were applied to compare diagnostic effects. Statistical significance was set at a two-sided *P* value of less than 0.05. The Attribute Fraction (AF) was used to calculate the proportion of CIN2+ lesions caused by specific HPV genotypes: AF= (contribution coefficient of target HPV genes × number of infections)/(CIN2+) ×100%. Based on the normal group, the relative risk (RR) of CIN1 and CIN2 was calculated as RR=AF (+)/AF (-).

## 3 Results

### 3.1 Characteristics of the study population

Of the 7,511 women were enrolled, 933 (12.42%) were diagnosed with ASC-US and categorized as the unvaccinated group. In this group, the average age was 47.42 ± 8.88 years, with around 70% having a junior middle school degree or below. The median ages of menarche and first pregnancy were 14 (13-16) and 23 (22-26) years, respectively. Almost all women in this group did not smoke or drink alcohol. In the vaccinated group, 754 women were induced, with an average age of 47.39 ± 8.92 years. There were no significant statistical differences between the two groups in terms of age, education level, marital status, alcohol consumption, smoking status, menarche age, and fertility history. Detailed results are shown in **Table 1**.

**Table 1:** Characteristics of the study population (n/%).

Characteristics	Group	Unvaccined group	Vaccined group	*χ^2^*	*P*
Age (yrs)	21~29	26 (2.78)	21 (2.78)	0.000	1.000
	30~39	157 (16.82)	127 (16.84)		
	≥40	750 (80.40)	606 (80.38)		
Level of education	Primary school and below	248 (26.58)	202 (26.79)	1.350	0.717
	Junior middle school	419 (44.91)	320 (42.44)		
	High school	101 (10.83)	86 (11.40)		
	≥University	165 (17.68)	146 (19.37)		
Marital status	Yes	918 (98.39)	744 (98.67)	0.140	0.707
	No	15 (1.61)	10 (1.33)		
Smoking	No	933 (100)	754 (100)	18.99	<0.01
	Yes	0 (0)	0 (0)		
Drinking	No	893 (95.71)	720 (95.49)	0.05	0.824
	Yes	40 (4.29)	34 (4.51)		
Age of menarche (yrs)	≤14	486 (52.09)	403 (53.44)	0.308	0.578
	>14	447 (47.91)	351 (46.56)		
Contraception measures	Sterilization Surgery	500 (53.59)	405 (53.71)	0.067	0.999
	Intrauterine Contraceptive Device	134 (14.36)	110 (14.58)		
	Oral Contraceptive Pills	1 (0.00)	1 (0.00)		
	Condom	75 (0.08)	59 (0.07)		
	No	223 (31.97)	179 (0.23)		
Age of the first pregnancy*	≤23	565 (60.56)	443 (59.06)	0.571	0.450
	>23	363 (39.44)	307 (40.94)		
Times of pregnancy	≤3	664 (71.16)	536 (71.08)	0.001	0.971
	>3	269 (28.84)	218 (28.92)		
Times of reproduction*	≤2	671 (72.15)	554 (73.86)	0.619	0.431
	>2	259 (27.85)	196 (26.14)		

*indicates missing data.

χ, Chi-square test.

### 3.2 Pathological diagnosis and attributable risk stratification analysis of CIN2+ by different HPV infection types in women with ASC-US

In the unvaccinated group, histopathology confirmed that 90.88% (848/933) of participants had a normal cervix. The proportions of participants with CIN1 and CIN2+ were 6.75% (63/933) and 2.35% (22/933), respectively. Among participants with ASC-US, the prevalence of HR-HPV was 59.27% (553/933). The prevalence of HR-HPV in participants with normal pathology, CIN1, and CIN2+ were 56.25% (477/848), 85.71% (54/63), and 100% (22/22), respectively. In the vaccinated group, histopathology confirmed that 94.16% (710/754) of participants had a normal cervix, while the proportions of participants with CIN1 or CIN2+ were 4.77% (36/754) and 1.06% (8/754), respectively (**Table 2**).

**Table 2:** The prevalence of infection with different HPV genotypes in women with ASC-US (n,%).

HPVgenotypes	Unvaccined group				Vaccined group			
	Normal	CIN1	CIN2+	Total	Normal	CIN1	CIN2+	Total
HPV16	103 (12.14)	23 (36.50)	10 (45.45)	136 (14.57)	–	–	–	–
HPV18	40 (4.41)	3 (4.76)	4 (18.18)	47 (5.03)	–	–	–	–
HPV31	33 (3.89)	6 (9.52)	2 (9.09)	41 (4.39)	30 (4.23)	2 (5.56)	2 (25.00)	34 (4.51)
HPV33	31 (3.65)	4 (6.34)	5 (22.72)	40 (4.28)	21 (2.96)	2 (5.56)	3 (37.50)	26 (3.45)
HPV52	100 (11.79)	11 (17.46)	3 (13.63)	114 (12.21)	78 (10.99)	6 (16.67)	2 (25.00)	86 (11.41)
HPV58	93 (10.96)	9 (14.28)	4 (18.18)	106 (11.36)	72 (10.15)	6 (16.67)	3 (37.50)	81 (10.75)
HPV51	72 (8.49)	8 (12.69)	2 (9.09)	82 (8.78)	54 (7.61)	2 (5.56)	2 (25.00)	58 (7.7)
HPV66	34 (4.00)	9 (14.28)	1 (4.54)	44 (4.71)	22 (3.10)	7 (19.45)	0 (0)	29 (3.85)
HPV68	28 (3.30)	1 (1.58)	1 (4.54)	30 (3.21)	22 (3.10)	1 (2.78)	1 (12.50)	24 (3.19)
HPV35	19 (2.25)	2 (3.17)	0	21 (2.25)	10 (1.41)	2 (5.56)	0 (0)	12 (1.6)
HPV39	36 (4.25)	2 (3.17)	2 (9.09)	40 (4.28)	28 (3.95)	1 (2.78)	0 (0)	29 (3.85)
HPV45	11 (1.30)	1 (1.58)	0	12 (1.28)	9 (1.27)	1 (2.78)	0 (0)	10 (1.33)
HPV59	29 (3.42)	1 (1.58)	0	30 (3.21)	22 (3.10)	0 (0)	0 (0)	22 (2.92)
HPV56	52 (6.13)	4 (6.34)	0	56 (6.00)	36 (5.08)	2 (5.56)	0 (0)	38 (5.04)
HR-HPV	477 (56.25)	54 (85.71)	22 (100.00)	553 (59.27)	339 (47.74)	27 (75.00)	8 (100.00)	374 (49.61)
Total	848 (90.88)	63 (6.75)	22 (2.36)	933 (100.00)	710 (94.16)	36 (4.77)	8 (1.06)	754 (100)

HPV, human papillomavirus; CIN 1/2/3, cervical intraepithelial neoplasia grade 1/2/3; “-”, negative.

In the unvaccinated group, the five most common HPV genotypes among normal participants were HPV16, 52, 58, 39 and 51. For those with CIN1, the top five HPV genotypes ranked by AF value were HPV16, 52, 58, 66, and 33, with HPV35 having the same AF as HPV39. Among participants with CIN2+, the five most common HPV types were HPV16, 33, 18, 58, and 31. In the vaccinated group, the incidence of HR-HPV infection increased with the severity of the pathological diagnosis. Among normal participants, the five most common types of HPV infections indicated by AF were HPV58, 52, 51, 31, and 56. The ranking of risks for CIN1, from high to low, was 52, 58, 66, 31, 33, 35,and 39. Among them, HPV types 33, 35, and 39 share the same rank. Among the CIN2+ population, the risk attribution of HPV from high to low was HPV33, 58, and 31. More details are shown in **Table 3**.

**Table 3:** Attributable risk analysis of different HPV types on CIN2+ (%,95%CI).

HPV genotype	Unvaccined group					Vaccined group				
	Normal	CIN1	CIN2+	RR(A)	RR(B)	Normal	CIN1	CIN2+	RR(A)	RR(B)
HPV16	0.11(0.09,0.13)	0.32(0.21,0.45)	0.44(0.32,0.58)	2.90	4.00	–	–	–	–	–
HPV18	0.02(0.01,0.04)	0.02(0.00,0.13)	0.13(0.02,0.39)	1.00	6.50	–	–	–	–	–
HPV33	0.02(0.01,0.03)	0.04(0.03,0.06)	0.22(0.07,0.53)	2.00	11.00	0.01(0.01,0.03)	0.02(0.00,0.15)	0.37(0.07,1.09)	2.00	37.00
HPV52	0.09(0.07,0.11)	0.11(0.10,0.12)	0	1.22	0	0.06(0.05,0.09)	0.13(0.04,0.32)	0	2.16	0
HPV58	0.08(0.06,0.10)	0.09(0.08,0.09)	0.13(0.09,0.17)	1.12	1.62	0.07(0.05,0.10)	0.11(0.03,0.28)	0.33(0.00,1.85)	1.57	4.71
HPV31	0.03(0.02,0.04)	0.02(0.01,0.03)	0.06(0.00,0.21)	0.66	2.00	0.03(0.02,0.04)	0.03(0.00,0.15)	0.16(0.00,0.92)	1.00	5.33
HPV35	0.00(0.00,0.01)	0.03(0.00,0.11)	0	–	0	0.00(0.00,0.01)	0.02(0.00,0.15)	0	–	0
HPV39	0.05(0.04,0.07)	0.03(0.01,0.09)	0	0.60	0	0.01(0.00,0.02)	0.02(0.00,0.15)	0	2.00	0
HPV45	0.00(0.00,0.01)	–	0	–	0	0.00(0.00,0.01)	–	0	–	0
HPV51	0.05(0.03,0.06)	–	0	–	0	0.05(0.03,0.07)	–	0	–	0
HPV56	0.03(0.02,0.05)	–	0	–	0	0.03(0.02,0.05)	–	0	–	0
HPV59	0.01(0.01,0.02)	–	0	–	0	0.02(0.01,0.03)	–	0	–	0
HPV66	0.02(0.01,0.03)	0.08(0.08,0.09)	0	4.00	0	0.01(0.00,0.02)	0.08(0.01,0.24)	0	8.00	0
HPV68	0.01(0.00,0.02)	0	0	0	0	0.01(0.01,0.03)	0	0	0	0

AF, Attribution score; RR, Relative risk; RR(A), AF_(CIN1)_/AF_(Normal)_; RR(B), AF_(CIN2+)_/AF_(Normal)_; “-”, negative.

### 3.3 The triaging value of different HPV genetypes in women with ASC-US

**Figure 2:**
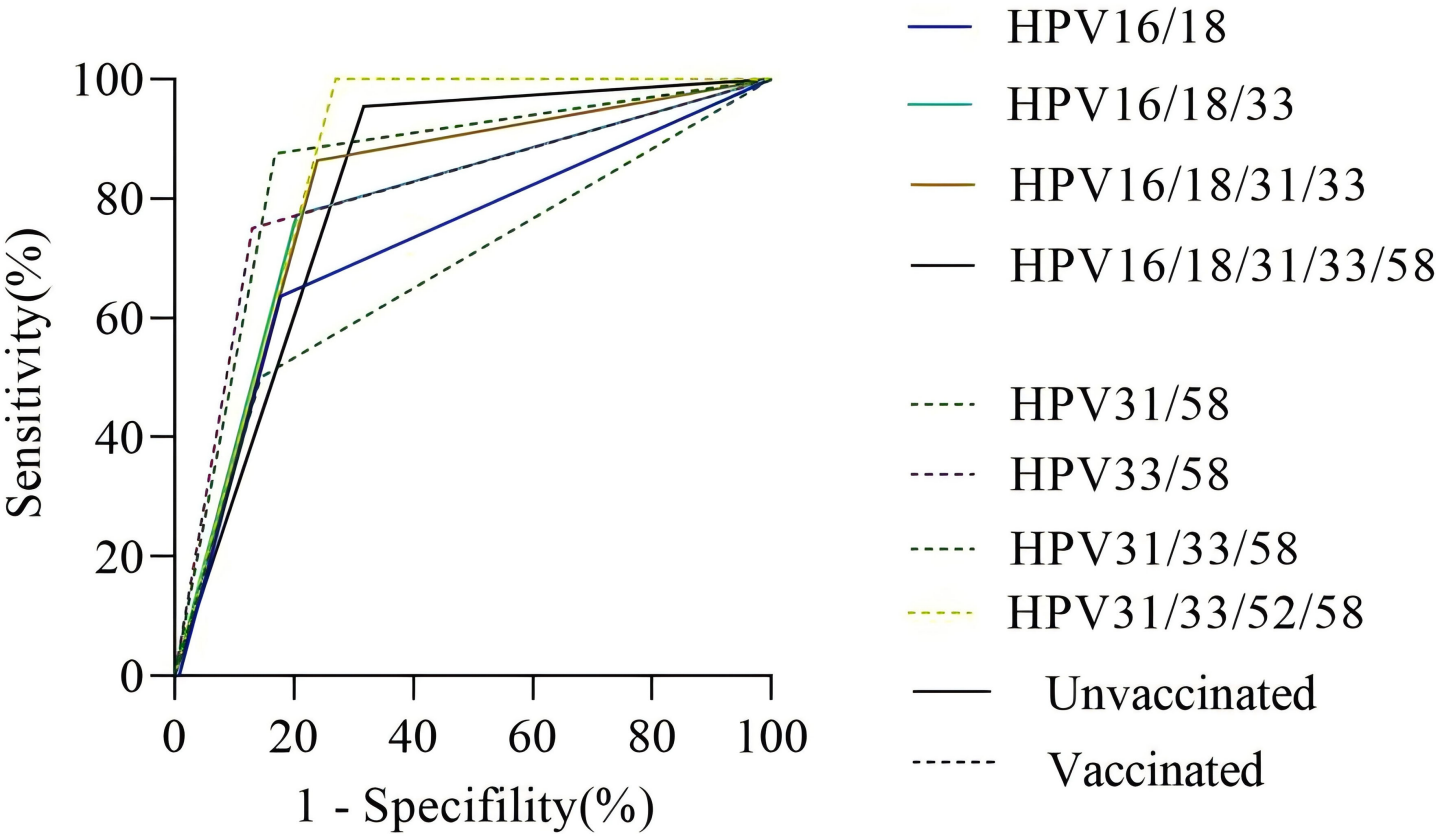
The ROC curve of different HR-HPV genotype combinations. Notes: The vertical axis represents the sensitivity of CIN2+ detection in different HPV genotype combinations, and the horizontal axis represents 1-specifility of CIN2+ detection in different HPV genotype combinations. The solid line represents the unvaccinated group, and the dotted line represents the vaccinated group.

**Figure 3:**
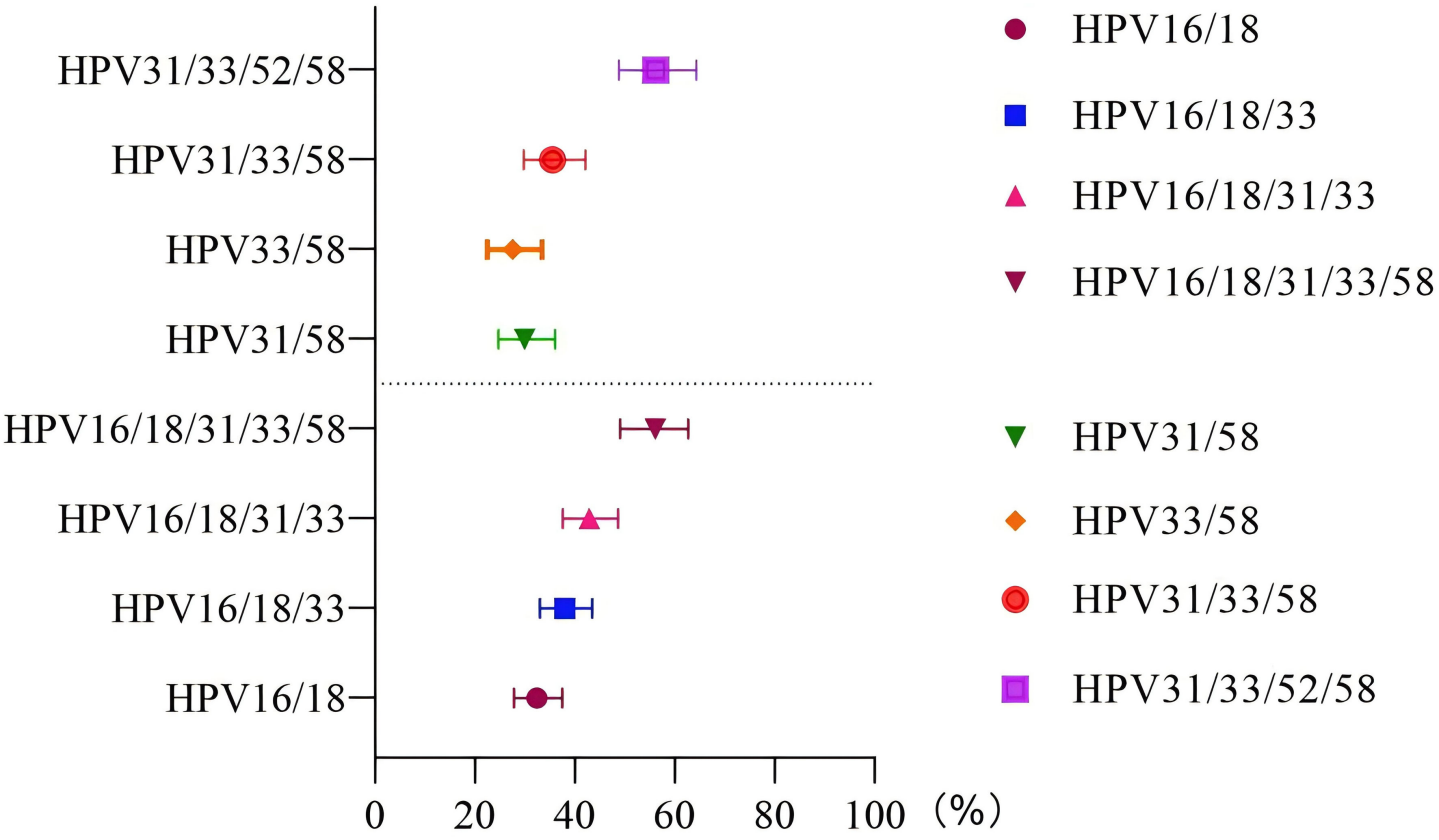
Referral rate in the unvaccinated and vaccinated groups. The vertical axis represents different HR-HPV genotype combination patterns, and the horizontal axis represents the probability of referral for colposcopy under different genotype combination patterns.

In the unvaccinated population, with CIN2+ histology of cervical lesions was the endpoint, the sensitivity and colposcopy referral rate of the combination HPV16/18 was the lowest compared to HPV16/18/31, HPV16/18/31/33, and HPV16/18/31/33/58 (63.63% vs. 77.27% vs. 86.36% vs. 95.45%; 32.37% vs. 37.97% vs. 42.86% vs. 56.06%). However the missed diagnosis rate of HPV16/18/31/33/58 (4.55%) was the lowest. In the vaccinated population, with CIN2+ histology of cervical lesions as the endpoint, the sensitivity and colposcopy referral rate of different combination models of HR-HPV increased with the inclusion of HPV33/58, HPV31/58, HPV31/33/58, and HPV31/33/52/58. However, the specificity of the combination HPV31/33/52/58 was the lowest compared to HPV33/58, HPV31/58, and HPV31/33/58 (72.92% vs. 85.52% vs. 86.99% or 83.11%). The ROC AUC of HPV33/58, HPV31/58, HPV31/33/58, and HPV31/33/52/58 were similar, while the referral rate of HPV33/58 was the lowest (27.54%), as shown in **Figures 2**, **3** and **Table 4**.

**Table 4:** The triaging effect of different HPV genotype on CIN2+ in women with ASC-US.

Group	HPV genotype	Sensitivity	Specificity*	PPV	NPV	Missed diagnosis rate	ROC AUC	Colposcopic referral rate*
Unvaccined group	HPV16/18	63.63 (40.65,82.80)	81.77* (79.11,84.23)	7.82 (5.67,10.69)	98.93 (98.16,99.38)	36.37 (17.20,59.35)	0.728 (0.698,0.756)	32.37* (27.80,37.47)
	HPV16/18/33	77.27 (54.63,92.17)	79.58* (76.81,82.15)	8.37 (6.58,10.60)	99.31 (98.53,99.68)	22.73 (7.83,45.37)	0.784 (0.756,0.810)	37.97* (33.01,43.47)
	HPV16/18/31/33	86.36 (65.08,97.09)	76.07* (73.16,78.80)	8.01 (6.64,9.64)	99.56 (98.77,99.84)	13.64 (2.91,34.92)	0.812 (0.786,0.836)	42.86* (37.57,48.67)
	HPV16/18/31/33/58	95.45 (77.15,99.88)	68.27* (65.14,71.29)	6.77 (5.98,7.65)	99.83 (98.92,99.97)	4.55 (0.12,22.85)	0.819 (0.792,0.843)	56.06* (49.09,62.66)
Vaccined group	HPV33	37.5 (8.52,75.51)	96.91^#^ (95.41,98.03)	11.53 (4.66,25.80)	99.31 (98.83,99.59)	62.50 (24.49,91.48)	0.672 (0.637,0.706)	6.95^#^ (4.54,10.18)
	HPV58	37.5 (8.52,75.51)	89.54^#^ (87.12,91.64)	3.70 (1.51,8.79)	99.25 (98.73,99.56)	62.50 (24.49,91.48)	0.635 (0.600,0.670)	21.66^#^ (17.2,26.92)
	HPV31	25.00 (3.18,65.08)	95.71^#^ (93.99,97.04)	5.88 (1.76,17.86)	99.16 (98.76,99.44)	75.00 (34.52,96.82)	0.604 (0.568,0.639)	9.09^#^ (6.29.12.70)
	HPV33/58	75.00 (34.91,96.81)	86.99* (84.37,89.32)	5.82 (3.82,8.77)	99.69 (98.98,99.90)	25.00 (3.19,65.09)	0.810 (0.780,0.837)	27.54* (22.48.33.40)
	HPV31/58	50.00 (15.70,84.29)	85.52* (82.79,87.97)	3.57 (1.78,7.03)	99.37 (98.76,99.68)	50.00 (84.30,15.71)	0.687 (0.643,0.711)	29.95* (24.66,36.03)
	HPV31/33/58	87.50 (47.34,99.68)	83.11* (80.22,85.73)	5.26 (3.92,7.01)	99.83 (99.00,99.97)	12.50 (0.32,52.64)	0.853 (0.826,0.878)	35.56* (29.77,42.14)
	HPV31/33/52/58	100.00 (63.05,100.00)	72.92* (69.58,76.08)	3.81 (3.40,4.26)	100.00	0	0.865 (0.838,0.888)	56.15* (48.81,64.28)

HPV, human papillomavirus; PPV, positive predictive value; NPV, negative predictive value; ROC AUC, the area under ROC curve; *, it means that there is *P* <0.05 between the combinational HPVgenotypes; #, it means that there is *P* <0.05 between the single HPV genotype.

## 4 Discussion

In regions with scarce resources and low hygiene levels, cervical cancer prevention and control are currently at a pivotal stage. This stage involves transforming vaccination strategies and confronting the dual responsibilities of advancing vaccine coverage and ensuring adequate screening for both vaccinated and unvaccinated groups. ASC-US is a common cytological abnormality in cervical cancer screening in the post-vaccine era, with histopathology that varies greatly (15). Due to the relatively limited diagnostic capabilities of cytologists, relying solely on TCT testing methods presents certain limitations. To optimize screening outcomes, introducing HPV testing can effectively compensate for the shortcomings of cytological screening.

Currently, there is a lack of substantial real-world datasets in China for reference purposes. We hypothesize that the bivalent vaccine was received by the study population to make a cautious estimation of post-immunization outcomes. In this study, the reporting rate of ASC-US among 7,511 rural women was about 12% in the unvaccinated group. The incidence rate of ASC-US in the population after vaccination was about 10%, similar to the range of 3.7-10% observed in Chinese women (16, 17). In the unvaccinated group, the study identified that the prevalence rate (59.27%) of HR-HPV in the ASC-US population was higher than the proportions reported by Zhang J (18) (43.79%) and Wang L (19) (49.76%) in rural Chinese areas, but lower than the figure reported by White C (20) (62.2%) in Ireland. This discrepancy may be due to differences in HPV infection rates among various regions. The CIN2+ is an important outcome endpoint in this study, with routine fertility-sparing treatments for early-stage cervical cancer including Loop Electrosurgical Excision Procedure (LEEP) and laparoscopic-assisted vaginal trachelectomy (21, 22). The CIN2+ detection rate among ASC-US individuals was 2.35% (22/933), which was similar to the rate reported by Ittiamornlert P (2.74%) (23) but lower than the rate reported by Tao X (5.5%) (15). This discrepancy might be attributed to the fact that our investigation carried out screening assessments within the general populace, whereas Tao X’s study enlisted participants through opportunistic screening procedures conducted at outpatient clinics. However, the detection rate of CIN2+ was only 1.06% (8/754) in the vaccinated group, significantly lower than in the unvaccinated group. Consistent with Teoh D’s (24) study, our findings showed that the probability of cervical precancerous lesions was lower in the vaccinated population compared to their unvaccinated counterparts.

In the unvaccined group, HPV16 had the highest infection rate and pathogenicity. HPV16 was the most prevalent genotype, with 44% of the risk of CIN2+ attributed to it (25). In addition to HPV16, the AF values for HPV33, 18, 58, and 31 were also high in CIN2+ cases. This contrasted with Li L’s study (26), where AF values were relatively higher for HPV16, 58, 52, 18, and 51, likely because our study population consisted of ASC-US individuals, whereas her research was conducted in the general population. Due to the protection provided by vaccination, the proportion of HPV genotypes has changed. In the vaccinated group, the AF values of HPV33, 58, and 31 ranked in the top three among individuals with CIN2+. HPV33, 58, and 31 should also be followed up in a short period. A similar study revealed that different types of HPV play distinct roles in cervical precancerous lesions (27). Previous studies (5, 28, 29) by domestic and foreign scholars analyzed the triage strategy of HPV16/18 and HR-HPV genotypes in ASC-US populations. A previous study found that the sensitivity and specificity of HPV16/18 genotyping in detecting CIN2+ lesions in 329 Chinese women with ASC-US were 82% and 91% (30). Another study in Shanxi province of China demonstrated that the sensitivity and specificity of HPV16/18/33/52/58 were 72.46% and 81.57%, respectively, for detecting CIN2+ lesions in women with ASC-US (31). In the current study, we evaluated the possibility of using a combination of the five most common HPV genotyping (HPV16/18/31/33/58). The sensitivity of HPV16/18 for ASC-US population in our study was similar to Li X’s findings (58.3%) (32). Our study also suggested that HPV16/18 (81.77%) saw the highest specificity in detecting CIN2+ in ASC-US compared to HPV16/18/33 (79.58%), HPV16/18/31/33 (76.07%), and HPV16/18/31/33/58 (68.27%), with significant difference. Moreover, the referral rate of HPV16/18 (32.37%) was the lowest, almost half of that of HPV16/18/31/33/58 (33.23%), which might avoid the waste of medical resources.

The incidence of HPV16/18 strains that lead to cervical cancer and its precursor lesions had declined with the onset of the vaccine era (33, 34). Studies conducted in India suggested that the HPV vaccine was more than 90% effective against HPV16/18 (35–37). In countries with high vaccine coverage, such as the United States and Australia, there had been a significant reduction in high-grade cervical lesions after the introduction of the HPV vaccine. In developing nations, the administration of bivalent vaccines had been extensively carried out among age-appropriate females under the auspices of local health policies. This measure contributed to reducing the future burden on both societal and familial levels and fostered improved female health. We assumed that the protection rate of post-bivalent vaccines would reach 100% in the vaccinated group. We evaluated the sensitivity, specificity, positive predictive value, and negative predictive value of other HPV genotype combinations excluding HPV16/18. Among single-genotype infections, HPV33 demonstrated relatively high specificity (96.91%) and the lowest referral rates (6.95%), demonstrating statistical significance against HPV31 and HPV58. It emerged as an excellent marker for assessing ASC-US triage within vaccinated populations. The sensitivity of HPV33/58 reached 75%, and the specificity was close to 90%, with a significant difference (*P*<0.05). In particular, the colposcopy referral rate (27.54%) was the lowest, and the difference was statistically different. Despite the unavoidable examination of colposcopy, the HPV vaccine will reduce the number of colposcopy referrals by 10% (38). HPV33/58 may be a new combination for the triage of ASC-US populations in the future. Consequently, this gene-specific genotyping test might help avoid unnecessary examinations and treatments.

This study has several limitations. Firstly, this hypothetical scenario disregards real-world variables affecting HPV vaccination’s impact, including coverage, compliance, and non-targeted HPV types. But the assumption grounded in comprehensive data of a large, real-world population that has undergone cervical cancer screening, can still offer valuable insights value for the triage of ASC-US women post-vaccination in the absence of comprehensive real-world research data on HPV vaccines. Secondly, only bivalent vaccines were considered, not quadrivalent and nine-valent vaccines. The bivalent vaccine was an economical option, and this research has carried out a cautious evaluation. The efficacy would be further improved if quadrivalent and nine-valent vaccines were employed.

## 5 Conclusion

In conclusion, in the unvaccinated group, HPV16, 18, 33, 58, and 31 genotypes require significant attention. The HPV16/18 genotyping strategy is a feasible for triaging participants with ASC-US in resource-limited areas. In the vaccinated group, HPV33, 58, and 31 genotypes require significant attention. The combination of HPV33/58 would be highly sensitive and specific for triaging the ASC-US population in the vaccinated group.

## Data Availability

The original contributions presented in the study are included in the article/supplementary material. Further inquiries can be directed to the corresponding author.

## References

[1] Chen T Wei M Liu Y Wang H Zhou W Bi Y. Rising mortality rate of cervical cancer in younger women in urban China. J Gen Intern Med. (2020) 35:593. doi: 10.1007/s11606-019-05174-5 31309406 PMC7018891

[2] Singh D Vignat J Lorenzoni V Eslahi M Ginsburg O Lauby-Secretan B. Global estimates of incidence and mortality of cervical cancer in 2020: a baseline analysis of the WHO Global Cervical Cancer Elimination Initiative. Lancet Glob Health. (2023) 11:e197–206. doi: 10.1016/S2214-109X(22)00501-0 PMC9848409 36528031

[3] Wang Y Gao S Wang Y Chen F Deng H Lu Y. The efficiency of type-specific high-risk human papillomavirus models in the triage of women with atypical squamous cells of undetermined significance. Cancer Manag Res. (2020) 12:5265–75. doi: 10.2147/CMAR.S254330 PMC7335862 32669875

[4] Frega A Pavone M Sesti F Leone C Bianchi P Cozza G. Sensitivity and specificity values of high-risk HPV DNA, p16/ki-67 and HPV mRNA in young women with atypical squamous cells of undetermined significance (ASCUS) or low-grade squamous intraepithelial lesion (LSIL). Eur Rev Med Pharmacol Sci. (2019) 23:10672–7. doi: 10.26355/eurrev20191219765 31858534

[5] Meijer C Snijders P. Human papillomavirus triage of women with atypical squamous cells of undetermined significance-reduction of overtreatment needed. JAMA Oncol. (2017) 3:1310–1. doi: 10.1001/jamaoncol.2017.1522 28654967

[6] Guo Z Jia MM Chen Q Chen HM Chen PP Zhao DM. Performance of different combination models of high-risk HPV genotyping in triaging chinese women with atypical squamous cells of undetermined significance. Front Oncol. (2019) 9:202. doi: 10.3389/fonc.2019.00202 31001472 PMC6456653

[7] Fokom-Defo V Dille I Fokom-Domgue J. Single dose HPV vaccine in achieving global cervical cancer elimination. Lancet Glob Health. (2024) 12:e360–1. doi: 10.1016/S2214-109X(24)00009-3 38365404

[8] Zhao FH Wu T Hu YM Wei LH Li MQ Huang WJ. Efficacy, safety, and immunogenicity of an Escherichia coli-produced Human Papillomavirus (16 and 18) L1 virus-like-particle vaccine: end-of-study analysis of a phase 3, double-blind, randomized, controlled trial. Lancet Infect Dis. (2022) 22:1756–68. doi: 10.1016/S1473-3099(22)00435-2 36037823

[9] Lehtinen M Lagheden C Luostarinen T Eriksson T Apter D Bly A. Human papillomavirus vaccine efficacy against invasive, HPV-positive cancers: population-based follow-up of a cluster-randomized trial. BMJ Open. (2021) 11:e50669. doi: 10.1136/bmjopen-2021-050669 PMC8719207 35149535

[10] Ren X Hao Y Wu B Jia X Niu M Wang K. Efficacy of prophylactic human papillomavirus vaccines on cervical cancer among the Asian population: A meta-analysis. Front Microbiol. (2022) 13:1052324. doi: 10.3389/fmicb.2022.1052324 36532442 PMC9755181

[11] Palmer TJ Kavanagh K Cuschieri K Cameron R Graham C Wilson A. Invasive cervical cancer incidence following bivalent human papillomavirus vaccination: a population-based observational study of age at immunization, dose, and deprivation. J Natl Cancer Inst. (2024) 116:857–65. doi: 10.1093/jnci/djad263 38247547

[12] Lin Z Liang X Su L Peng W Chen H Fang Y. Coverage with the first dose of human papillomavirus vaccination among females aged 9-50 years in shenzhen, China: A surveillance based on administrative health records in 2023. Vaccines (Basel). (2024) 12:75–6. doi: 10.3390/vaccines12010075 PMC10818281 38250888

[13] Zhao XL Hu SY Hu JW Wang HH Wen TM Feng YS. Tackling barriers to scale up human papillomavirus vaccination in China: progress and the way forward. Infect Dis Poverty. (2023) 12:86. doi: 10.1186/s40249-023-01136-6 37735709 PMC10512493

[14] Reich O Regauer S Marth C Schmidt D Horn LC Dannecker C. Precancerous lesions of the cervix, vulva and vagina according to the 2014 WHO classification of tumors of the female genital tract. Geburtshilfe Frauenheilkd. (2015) 75:1018–20. doi: 10.1055/s-0035-1558052 PMC4629991 26556904

[15] Tao X Austin RM Yu T Zhong F Zhou X Cong Q. Risk stratification for cervical neoplasia using extended high-risk HPV genotyping in women with ASC-US cytology: A large retrospective study from China. Cancer Cytopathol. (2022) 130:248–58. doi: 10.1002/cncy.v130.4 34874615

[16] Li B Dong L Wang C Li J Zhao X Dong M. Analysis of the related factors of atypical squamous cells of undetermined significance (ASC-US) in cervical cytology of post-menopausal women. Front Cell Infect Microbiol. (2023) 13:1123260. doi: 10.3389/fcimb.2023.1123260 36875525 PMC9978476

[17] Tao X Zhang H Wang L Pan Q Ji S Zhou X. Atypical squamous cells of undetermined significance cervical cytology in the Chinese population: Age-stratified reporting rates, high-risk HPV testing, and immediate histologic correlation results. Cancer Cytopathol. (2021) 129:24–32. doi: 10.1002/cncy.v129.1 32697438

[18] Zhang J Zhao Y Dai Y Dang L Ma L Yang C. Effectiveness of high-risk human papillomavirus testing for cervical cancer screening in China: A multicenter, open-label, randomized clinical trial. JAMA Oncol. (2021) 7:263–70. doi: 10.1001/jamaoncol.2020.6575 PMC7774051 33377903

[19] Wang L Song Q Liu Y Ou Q. ThinPrep cytologic test combined with HPV typing to evaluate the degree of cervical diseases and the relationship between HPV typing and the pathological results of patients with atypical squamous cells of undetermined significance: a diagnostic test. Transl Cancer Res. (2022) 11:3277–86. doi: 10.21037/tcr-22-2026 PMC9552063 36237241

[20] White C Bakhiet S Bates M Keegan H Pilkington L Ruttle C. Triage of LSIL/ASC-US with p16/Ki-67 dual staining and human papillomavirus testing: a 2-year prospective study. Cytopathology. (2016) 27:269–76. doi: 10.1111/cyt.2016.27.issue-4 26932360

[21] Pavone M Goglia M Scambia G Querleu D Akladios C Lecointre L. Laparoscopic-assisted vaginal trachelectomy with prophylactic cerclage: A safe fertility-sparing treatment for early stage cervical cancer. Ann Surg Oncol. (2024) 31:1804–5. doi: 10.1245/s10434-023-14737-0 38071714

[22] Frega A Santomauro M Sesti F Di Giuseppe J Colombrino C Marziani R. Preterm birth after loop electrosurgical excision procedure (LEEP): how cone features and microbiota could influence the pregnancy outcome. Eur Rev Med Pharmacol Sci. (2018) 22:7039–44. doi: 10.26355/eurrev20181016176 30402872

[23] Ittiamornlert P Jareemit N Phianpiset R Kuljarusnont S Hanamornroongruang S Horthongkham N. High-risk human papillomavirus genotyping in women with atypical squamous cells of undetermined significance. Sci Rep. (2023) 13:12134. doi: 10.1038/s41598-023-39206-2 37495771 PMC10372087

[24] Teoh D Nam G Aase DA Russell R Melton GB Kulasingam S. Test performance of cervical cytology among adults with vs without human papillomavirus vaccination. JAMA Netw Open. (2022) 5:e2214020. doi: 10.1001/jamanetworkopen.2022.14020 35612854 PMC9133945

[25] Del MA Adcock R Carozzi F Gillio-Tos A De Marco L Girlando S. Human papilloma virus genotyping for the cross-sectional and longitudinal probability of developing cervical intraepithelial neoplasia grade 2 or more. Int J Cancer. (2018) 143:333–42. doi: 10.16462/j.cnki.zhjbkz.2017.01.021 PMC6099271 29453769

[26] Li L Jiang M Li T Yin J Feng R Dong L. Absolute risk and attributable fraction of type-specific human papillomavirus in cervical cancer and precancerous lesions-A population-based study of 6286 women in rural areas of China. J Clin Med. (2022) 11:6483–95. doi: 10.3390/jcm11216483 PMC9655002 36362711

[27] Zhao XL Hu SY Zhang Q Dong L Feng RM Han R. High-risk human papillomavirus genotype distribution and attribution to cervical cancer and precancerous lesions in a rural Chinese population. J Gynecol Oncol. (2017) 28:e30. doi: 10.3802/jgo.2017.28.e30 28541628 PMC5447139

[28] Wright TJ Stoler MH Parvu V Yanson K Cooper C Andrews J. Risk detection for high-grade cervical disease using Onclarity HPV extended genotyping in women, ≥21 years of age, with ASC-US or LSIL cytology. Gynecol Oncol. (2019) 154:360–7. doi: 10.1016/j.ygyno.2019.05.012 31160073

[29] Saccucci M Franco EL Ding L Bernstein DI Brown D Kahn JA. Non-vaccine-type human papillomavirus prevalence after vaccine introduction: no evidence for type replacement but evidence for cross-protection. Sex Transm Dis. (2018) 45:260–5. doi: 10.1097/OLQ.0000000000000731 PMC7193949 29465705

[30] Lin CQ Cui JF Zhang X Pan QJ Chen W Qiao YL. Human papillomavirus genotyping to predict the risk of cervical precancerous lesions or cancer in women with minor abnormal cytology in China. Acta Cytol. (2015) 59:405–11. doi: 10.1159/000441290 26565687

[31] Wei S Tao Z Tie-jun Z Gen-ming Z. The epidemiological situation of human papillomavirus infection among women in China. Chin J Pract Gynecology Obstetrics. (2017) 21:89–93. doi: 10.16462/j.cnki.zhjbkz.2017.01.021

[32] Li X Kang C Kong W Xu C Song Y Xing K. Clinical significance of shunt of different subtypes of high-risk human papillomavirus in atypical squamous cell patients with unknown cervical cytology. Chin J Pract Gynecology Obstetrics. (2023) 39:749–52. doi: 10.19538/j.fk2023070116

[33] Abbas M de Jonge J Bettendorf O. Prevalence of high-risk HPV subtypes and efficacy of the HPV vaccine in preventing cervical epithelial lesions: survey and insights from a german study. Life (Basel). (2023) 13:1637–46. doi: 10.3390/life13081637 PMC10455871 37629494

[34] Covert C Ding L Brown D Franco EL Bernstein DI Kahn JA. Evidence for cross-protection but not type-replacement over the 11 years after human papillomavirus vaccine introduction. Hum Vaccin Immunother. (2019) 15:1962–9. doi: 10.1080/21645515.2018.1564438 PMC6746493 30633598

[35] Basu P Malvi SG Joshi S Bhatla N Muwonge R Lucas E. Vaccine efficacy against persistent human papillomavirus (HPV) 16/18 infection at 10 years after one, two, and three doses of quadrivalent HPV vaccine in girls in India: a multicenter, prospective, cohort study. Lancet Oncol. (2021) 22:1518–29. doi: 10.1016/S1470-2045(21)00453-8 PMC8560643 34634254

[36] Setiawan D Nurulita NA Khoirunnisa SM Postma MJ. The clinical effectiveness of one-dose vaccination with an HPV vaccine: A meta-analysis of 902,368 vaccinated women. PloS One. (2024) 19:e290808. doi: 10.1371/journal.pone.0290808 PMC10769028 38180991

[37] Zhao F Jastorff A Hong Y Hu S Chen W Xu X. Safety of AS04-HPV-16/18 vaccine in Chinese women aged 26 years and older and long-term protective effect in women vaccinated at age 18-25 years: A 10-year follow-up study. Asia Pac J Clin Oncol. (2023) 19:458–67. doi: 10.1111/ajco.13833 36101936

[38] Pesola F Rebolj M Leeson S Dunk L Pickford L Gjini A. Introducing human papillomavirus (HPV) primary testing in the age of HPV vaccination: projected impact on colposcopy services in Wales. BJOG. (2021) 128:1226–35. doi: 10.1111/1471-0528.16610 PMC8246959 33247993

